# Unveiling the Molecular Mechanism of n-Bromobutane Synthesis Experiment: A DFT Study for Undergraduate Organic Chemistry Teaching

**DOI:** 10.3390/molecules31101690

**Published:** 2026-05-16

**Authors:** Xiaobing Lan, Yong Zhao, Dongyi Hong, Rongkun Ouyang, Jiawei Li, Jun Chen

**Affiliations:** Hunan Provincial Key Laboratory of Xiangnan Rare-Precious Metals Compounds Research and Application, School of Chemistry and Environmental Science, Xiangnan University, Chenzhou 423000, China; 19973466638@163.com (Y.Z.); 15673390263@163.com (D.H.); 17378206756@163.com (R.O.); ljw2020upup@163.com (J.L.)

**Keywords:** organic chemistry, teaching experiment, synthesis of n-bromobutane, reaction mechanism, DFT study

## Abstract

The synthesis of n-bromobutane from n-butanol is a classic undergraduate organic chemistry experiment, primarily intended to illustrate the bimolecular nucleophilic substitution (S_N_2) mechanism. However, this experiment is commonly plagued by low yields and the formation of byproducts (e.g., n-butene and di-n-butyl ether), which confuse students. To reveal the molecular origin of these competitive pathways, this study employs density functional theory (DFT) calculations to systematically investigate the reaction mechanism under acid catalysis. Four potential reaction pathways were explored: S_N_2 substitution, E2 elimination, intermolecular etherification, and a high-energy E2 pathway. The computational results indicate that the S_N_2 pathway to n-bromobutane is kinetically and thermodynamically favorable due to its low energy barrier. In contrast, the E2 elimination pathway possesses a higher energy barrier (18.8 kcal/mol vs. 13.5 kcal/mol for S_N_2), explaining why elevated temperatures favor the formation of n-butene. Moreover, the etherification pathway was found to be the most energetically demanding, consistent with the trace amounts of di-n-butyl ether observed experimentally. These findings provide a quantitative molecular-level rationale for the strict temperature control and standardized reagent addition sequences in the laboratory protocol. By visualizing the potential energy surfaces, this computational approach bridges the gap between theoretical mechanism and practical operation, offering a valuable pedagogical tool for enhancing student understanding.

## 1. Introduction

Experimental teaching in fundamental organic chemistry serves as a critical bridge connecting theoretical knowledge with practical skills, occupying an irreplaceable and central role in the curriculum [1,2,3,4,5]. Among these classic experiments, the synthesis of n-bromobutane stands out as one of the most representative cases, primarily designed to illustrate the fundamental bimolecular nucleophilic substitution (S_N_2) mechanism [6,7,8]. This experiment is particularly instructive as it encompasses key operational techniques, including reflux, distillation, and liquid–liquid extraction, while also demonstrating the delicate balance between substitution and elimination reactions [9,10,11]. Furthermore, it allows students to deeply understand the core principles of how reaction temperature and reagent addition sequences regulate reaction selectivity. Aligned with theoretical topics including haloalkane synthesis and the competition between nucleophilic substitution and elimination, this experiment serves as a vital link between theory and laboratory practice.

Despite being a mature and classic experiment, students frequently encounter issues such as low yields and the formation of dark-colored byproducts caused by carbonization. Unlike esterification [12,13,14] or etherification [15] experiments, the unique challenge in n-bromobutane synthesis lies in the following competitive processes: (1) the desired S_N_2 pathway versus the E2 elimination pathway, and (2) the interference caused by the oxidation of HBr by concentrated sulfuric acid. The conventional protocol utilizes NaBr and concentrated H_2_SO_4_ to generate HBr in situ, forming a complex aqueous reaction mixture. In this environment, the strong acidic conditions can easily promote the dehydration of n-butanol to n-butene if the temperature is not rigorously controlled. Furthermore, the optimal balance between sufficient protonation (to convert –OH into –OH_2_^+^) and avoiding excessive acidity (which leads to carbonization via oxidation or excessive dehydration) often remains a “black box” for students. Traditional teaching frequently relies on rote memorization of protocols; merely asking students to memorize the addition sequence leaves them unable to understand “why the order matters if everything ends up mixed in the flask.” Similarly, simply emphasizing temperature limits leaves them puzzled as to “why temperature changes the product type.” Consequently, some students may ignore specified protocols, leading to experimental failure.

From our knowledge, the core issue stems from the competitive nature of reaction pathways under acidic conditions. Under acidic conditions, n-butanol is protonated to form an oxonium ion, a crucial step that converts the hydroxyl group into a viable leaving group. This intermediate then faces a kinetic competition: attack by the bromide nucleophile (S_N_2) versus abstraction of a β-hydrogen by a base (E2). While traditional teaching emphasizes procedural steps, DFT calculations can illuminate this “black box” by quantifying the energy landscapes of these competitive pathways. We believe that only by helping students deeply understand the reaction mechanism, allowing them to deduce the reasons for operational requirements and the consequences of violations on their own, can we truly achieve knowledge transfer.

Recently, DFT calculations have offered a visual and quantitative approach to demystify these processes and have been widely applied in organic chemistry research and teaching [16,17,18,19,20]. While recent studies have utilized DFT to optimize the teaching of esterification [14] and ether synthesis [15], few studies have employed DFT calculations to analyze the synthesis mechanism of n-bromobutane. Herein, we extend the computational analysis specifically to the halogenation mechanism, highlighting the unique role of in situ acid concentration and nucleophile strength in determining product distribution. Using Gaussian 09, we systematically investigated the Brønsted acid-catalyzed conversion of n-butanol to n-bromobutane. By computationally elucidating the energy barriers of the main and side reactions, we aim to establish a robust theoretical framework that turns experimental failures into teachable moments for understanding reaction selectivity.

## 2. Results and Discussion

### 2.1. Procedure of n-Bromobutane Synthesis Experiment

**Figure 1:**
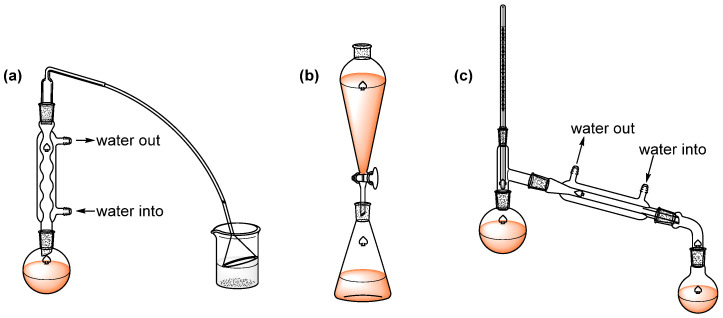
Schematic of the n-bromobutane synthesis experiment. (**a**) Reflux reaction setup; (**b**) liquid–liquid extraction with a separatory funnel; (**c**) distillation purification.

**Figure 2:**
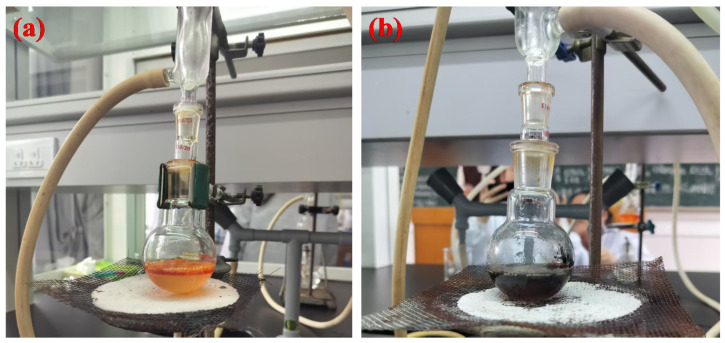
Experimental phenomena of the n-bromobutane synthesis: (**a**) normal reaction mixture; (**b**) carbonized mixture caused by overheating.

The experimental protocol and corresponding apparatus are illustrated in Figure 1, and the typical reaction phenomena are presented in Figure 2. The detailed experimental procedure is as follows:

(1) Reagent mixing: Add 5 mL of water to a 50 mL round-bottom flask, followed by the slow addition of 7 mL of concentrated sulfuric acid (stirring continuously to prevent local overheating). After the solution has cooled, add 4.6 mL of n-butanol, 7.5 g of anhydrous NaBr, and boiling chips. Upon completion of reagent addition, assemble the apparatus using a 5% NaOH solution as the absorbent in preparation for the subsequent heating process.
(2) Reflux reaction: Heat gently to maintain a gentle reflux for 30–40 min (Figure 1a). Afterward, stop heating and allow the mixture to cool. Reconfigure the apparatus for distillation (if additional boiling chips are needed, add them only after cooling to prevent bumping), and heat again to distill off all the crude n-bromobutane product, thereby completing the reaction stage.
(3) Distillation and separation: Transfer the distillate into a separatory funnel (Figure 1b). First, wash with an equal volume of water; after shaking and allowing the layers to separate, isolate the organic layer. Next, wash with an equal volume of concentrated sulfuric acid, followed by another wash with an equal volume of water to remove impurities step-by-step. The liquid obtained from the above washing steps is then washed with a 10% sodium carbonate solution to adjust the pH, followed by a final wash with an equal volume of water. Transfer the treated liquid into a dry conical flask and dry with anhydrous calcium chloride for approximately 0.5 h until the liquid becomes clear and transparent. Finally, transfer the dried liquid into a small round-bottom flask for distillation (Figure 1c), collecting the fraction at 99–103 °C to obtain the purified n-bromobutane product.

### 2.2. Reaction Equations and Mechanism Analysis

**Figure 3:**
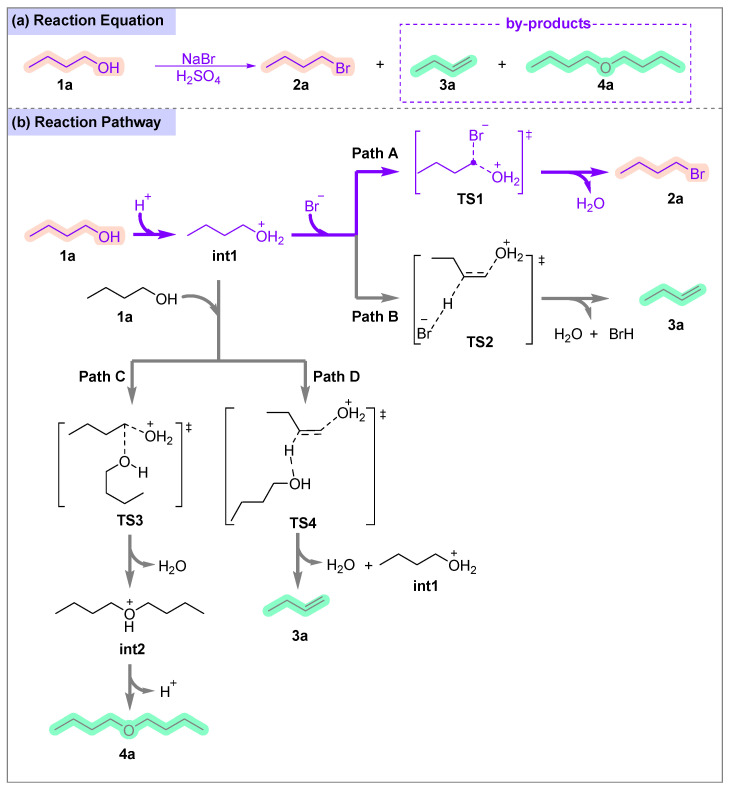
Reaction model and mechanism. (**a**) Reaction Equation. (**b**) Reaction pathway.

The Brønsted acid-catalyzed conversion of n-butanol (**1a**) to n-bromobutane (**2a**) is shown in Figure 3a, with the general mechanistic pathway depicted in Figure 3b.

The reaction initiates with protonation of the hydroxyl group by H^+^ (from H_2_SO_4_) to form oxonium intermediate **int1**, which converts the poor leaving group (–OH) into a good leaving group (–OH_2_^+^). The nucleofugality index Λ, as defined by Domingo and his co-worker [21], quantifies the leaving group ability. For neutral -OH, Λ is very low (~0.2 eV), whereas protonated –OH_2_^+^ exhibits a dramatically higher Λ (~5–6 eV). This explains why protonation is essential to convert the poor –OH leaving group into an excellent –OH_2_^+^ leaving group in this system. From **int1**, four competitive pathways proceed: Path A (S_N_2 Substitution): The bromide ion (Br^−^) performs a backside attack on the α-carbon of **int1**, passing through transition state structure **TS1**, resulting in the formation of n-bromobutane. Path B (E2 Elimination): Br^−^ acts as a base, abstracting a β-hydrogen from **int1** via transition state structure **TS2**, leading to the formation of 1-butene. Path C (Intermolecular Etherification): A neutral molecule of n-butanol (**1a**) acts as the nucleophile to attack the α-carbon of **int1**, proceeding through transition state structure **TS3** to form di-n-butyl ether. Path D (High-Energy E2): A concerted elimination pathway with severe geometric distortion, proceeding via transition state structure **TS4**.

### 2.3. Reaction Potential Energy Surface Analysis

**Figure 4:**
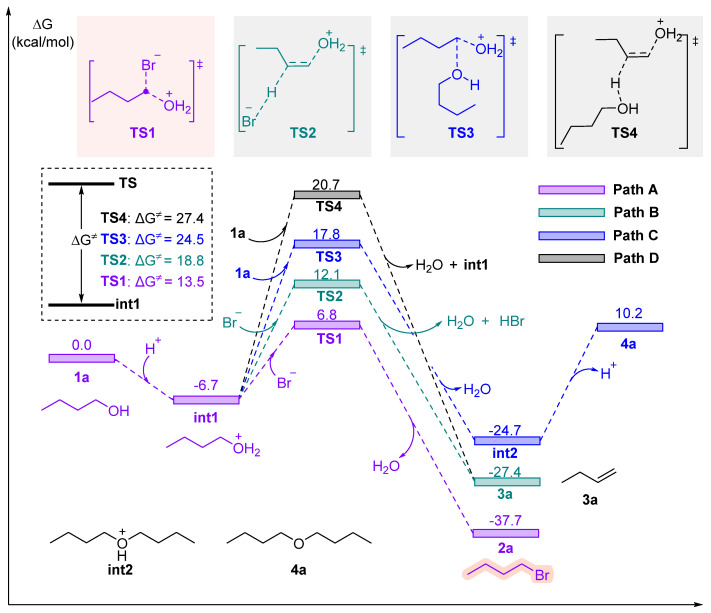
The potential energy profiles of the competitive reaction pathways.

In chemical reactions, a transition state (TS) represents the highest-energy structure along the reaction coordinate, where bonds are partially broken and formed. The activation Gibbs free energy (ΔG^≠^) determines the kinetic feasibility of a pathway: the lower the barrier, the faster the reaction. Thus, we located and compared the TSs for four competing pathways to rationalize the experimentally observed product distribution. The potential energy surface for the reaction pathways was calculated using DFT, with the results shown in Figure 4. The reaction initiates with the protonation of n-butanol (**1a**, ΔG = 0.0 kcal/mol) to form **int1** (ΔG = −6.7 kcal/mol). This step is exothermic and spontaneous, facilitating rapid reaction activation. It quantitatively reveals the competition between these pathways. Path A (Main Reaction): The S_N_2 pathway exhibits the lowest activation Gibbs free energy of 13.5 kcal/mol (relative to the protonated intermediate **int1**). The reaction is highly exothermic (ΔG = −37.7 kcal/mol), confirming that n-bromobutane is the thermodynamically favored product. Path B (Side Reaction): The E2 elimination pathway has a higher energy barrier (18.8 kcal/mol). Although the product (n-butene) is thermodynamically stable, the higher kinetic barrier explains why this pathway is suppressed at lower temperatures. However, the relatively small difference in energy barriers (~5.3 kcal/mol) indicates that elevated temperatures will significantly increase the proportion of elimination product. Path C (Etherification): This bimolecular pathway possesses the highest energy barrier (24.5 kcal/mol), making it kinetically unfavorable. This correlates perfectly with the experimental observation that di-n-butyl ether is formed only in negligible traces. Path D: The most unfavorable **TS4** has an activation Gibbs free energy of 27.4 kcal/mol, rendering this pathway non-competitive under standard conditions.

The reaction potential energy surface clearly reveals the competitive relationship between substitution, elimination, and etherification initiated by the same intermediate, explaining the product distribution under different conditions: Under low temperature and short heating times, the S_N_2 pathway dominates to produce n-bromobutane; conversely, high temperatures and prolonged heating tend to trigger E2 elimination to form alkenes, while the strong dehydrating property of concentrated sulfuric acid can lead to system carbonization. The experimental procedure of adding water before concentrated sulfuric acid and adding reagents slowly is designed not only to avoid the dangers associated with the heat of dilution but also to prevent local carbonization. It should be noted that recomputing the Gibbs free energies at 373 K (reflux temperature) gave nearly identical energy differences between the competing TSs (see Figure S1, Supporting Information), confirming that our conclusions are robust across the relevant temperature range.

### 2.4. Electrostatic Potential and Structural Analysis

**Figure 5:**
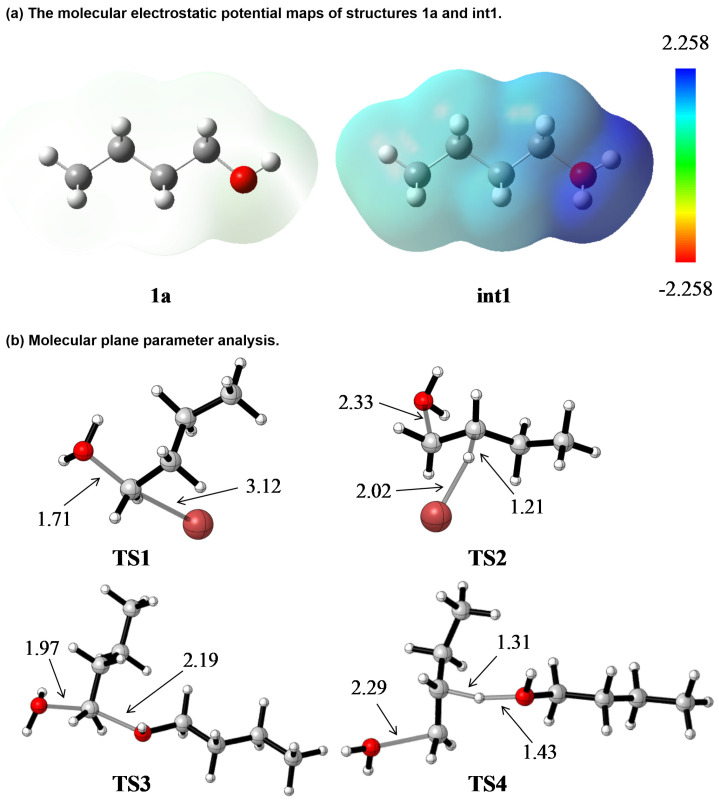
(**a**) The molecular electrostatic potential maps of structures **1a** and **int1** [15]. (**b**) Molecular plane parameter and key geometric analysis of transition state structures **TS1**–**TS4**.

The molecular electrostatic potential maps for n-butanol (**1a**) and the protonated intermediate **int1** further corroborate the mechanistic preferences, and the results are shown in Figure 5a. The protonated intermediate **int1** displays a significantly more positive electrostatic potential on the α-carbon compared to neutral n-butanol (**1a**), highlighting its susceptibility to nucleophilic attack by Br^−^.

The structural analysis of the transition states was also performed, and the results are shown in Figure 5b. It provides geometric insights into the energy barriers: **TS1** displays a classic Walden inversion geometry, with the Br–C(α) bond forming simultaneously as the C(α)–OH_2_^+^ bond breaks. According to recent MEDT studies [21,22,23], the transition states of S_N_2 reactions are characterized by complete cleavage of the C-LG bond, absence of Nu-C bond formation, and a carbocationic central carbon. Our calculated **TS1** (Figure 5b) fully supports this finding. In our results, the C(α)–OH_2_^+^ distance is 1.71 Å (broken), the Br–C(α) distance is 3.12 Å (not yet formed) and the C(α) atom adopts a sp^2^-hybridized configuration, consistent with a carbocation-like transition state. **TS2** exhibits a coplanar arrangement where the Br^−^ abstracts the β-hydrogen concurrent with the departure of the water molecule. The geometric constraints of this transition state contribute to its higher energy compared to **TS1**. **TS3** involves a crowded transition state with two bulky butyl groups, leading to steric repulsion that raises the energy barrier. **TS4** represents an E2 transition state with severe steric repulsion that raises the energy barrier, resulting in a remarkably high energy barrier (27.4 kcal/mol), making this pathway completely non-competitive under standard experimental conditions. No favorable electrostatic interaction or geometric relaxation is observed in **TS4**, further confirming its inaccessibility in the reaction system.

## 3. Materials and Methods

All DFT calculations were performed using the Gaussian 09 software package [24]. The geometries of all reactants, intermediates, transition states (TS), and products were fully optimized using the B3LYP [25,26,27] functional with the 6-31G(d,p) basis set. Grimme’s D3 [28] dispersion correction was applied to all calculations to account for van der Waals interactions. Frequency calculations were conducted to confirm the nature of the stationary points: minima (showing no imaginary frequencies) and transition states (exhibiting a single imaginary frequency corresponding to the reaction coordinate). Intrinsic Reaction Coordinate (IRC) [29] calculations were performed to verify that the transition states connected the designated reactants and products. To enhance the accuracy of energetic evaluations, single-point energy calculations were carried out on the optimized structures using the larger B3LYP/6-311++G(d,p) basis set. The solvation effects were modeled using the SMD [30] continuum solvent model with parameters corresponding to n-butanol. Molecular structures were visualized using the CYLview program (version 2.0) [31]. All of the optimized Cartesian coordinates of species involved in the reactions are available in the Supporting Information (SI).

## 4. Conclusions

In summary, this DFT study has successfully mapped the potential energy landscape of the n-bromobutane synthesis reaction. Our calculations confirm that the S_N_2 pathway is the dominant mechanism due to its low kinetic barrier and high thermodynamic stability. An energy difference of approximately 5 kcal/mol renders the higher-energy E2 transition state essentially non-competitive under mild conditions, but this barrier can be overcome at elevated temperatures, explaining why the elimination product forms preferentially under harsh conditions. These computational results provide a rigorous scientific justification for the empirical rules taught in the laboratory. The necessity for “gentle reflux” is no longer an arbitrary instruction but a kinetic requirement to suppress the E2 pathway. Furthermore, the calculated high barrier for etherification explains the experimental insignificance of this side reaction. By integrating these computational findings into the teaching curriculum, educators can move beyond procedural memorization. This approach allows students to visualize the energy barriers their molecules must overcome, transforming the n-bromobutane experiment from a simple synthesis into a profound lesson on the intimate relationship between reaction conditions and molecular mechanism.

## Data Availability

The original contributions presented in this study are included in the article. Further inquiries can be directed to the corresponding authors.

## Supplementary Materials

The following supporting information can be downloaded at https://www.mdpi.com/article/10.3390/molecules31101690/s1, Optimized Cartesian coordinates of intermediates and transition states involved in Figure 4. Figure S1. The potential energy profiles of the competitive reaction pathways (at 373 K). A step-by-step hands-on tutorial for the S_N_2 pathway (Path A). Refs. [32,33,34] are cited in the Supplementary Materials.
