# Supplementary material for: Unveiling the Molecular Mechanism of n-Bromobutane Synthesis Experiment: A DFT Study for Undergraduate Organic Chemistry Teaching
Source: Molecules. 2026 May 16;31(10):1690. doi: 10.3390/molecules31101690 (PMC13210258; doi:10.3390/molecules31101690)
Supplement: Supplementary file 1 [file molecules-31-01690-s001.zip › molecules-4290350-supplementary.pdf]

Supporting Information

Unveiling the Molecular Mechanism of n-Bromobutane Synthesis Experiment: A DFT Study for Undergraduate Organic Chemistry Teaching

Xiaobing Lan *, Yong Zhao, Dongyi Hong, Rongkun Ouyang, Jiawei Li and Jun Chen *

Hunan Provincial Key Laboratory of Xiangnan Rare-Precious Metals Compounds Research and Application, School of Chemistry and Environmental Science, Xiangnan University, Chenzhou 423000, China

* Correspondence: xblan@xnu.edu.cn (X.L.); chenjun4174@126.com (J. C.)

1. Optimized Cartesian coordinates……………………….……………………2

2. The potential energy profiles of the competitive reaction pathways (at 373 K)......12

3. A step-by-step hands-on tutorial for the S_N2 pathway (Path A)..............................13

1. Optimized Cartesian coordinates

1a

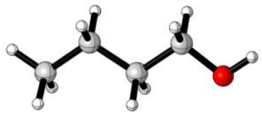

0 1

C	-3.66076500	-0.48004600	-0.00000600
H	-4.62057000	0.04605100	-0.00001100
H	-3.63242200	-1.12647200	0.88420500
H	-3.63241800	-1.12648200	-0.88421000
C	-2.48604600	0.50381600	-0.00000900
H	-2.55374600	1.15919400	0.87861800
H	-2.55374100	1.15918400	-0.87864400
C	-1.12622300	-0.21243100	-0.00000200
H	-1.04640900	-0.86203900	-0.87934100
H	-1.04641300	-0.86202900	0.87934600
C	0.02521700	0.78102000	-0.00000400
H	0.02724300	1.41245500	0.88977900
H	0.02724800	1.41244500	-0.88979500
Br	1.78623200	-0.13618500	0.00000500

2a

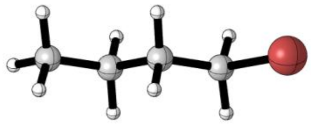

0 1

C	-3.66076500	-0.48004600	-0.00000600
H	-4.62057000	0.04605100	-0.00001100
H	-3.63242200	-1.12647200	0.88420500

H	-3.63241800	-1.12648200	-0.88421000
C	-2.48604600	0.50381600	-0.00000900
H	-2.55374600	1.15919400	0.87861800
H	-2.55374100	1.15918400	-0.87864400
C	-1.12622300	-0.21243100	-0.00000200
H	-1.04640900	-0.86203900	-0.87934100
H	-1.04641300	-0.86202900	0.87934600
C	0.02521700	0.78102000	-0.00000400
H	0.02724300	1.41245500	0.88977900
H	0.02724800	1.41244500	-0.88979500
Br	1.78623200	-0.13618500	0.00000500

3a

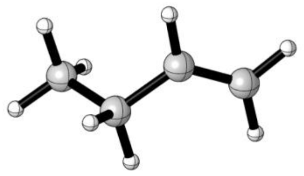

0 1

C	1.72450800	-0.25329700	-0.29276600
H	2.63928200	0.34835700	-0.28588800
H	1.51626800	-0.54776000	-1.32638700
H	1.92021500	-1.16696400	0.27984400
C	0.54062600	0.52880100	0.30170800
H	0.36823600	1.44425500	-0.27735700
H	0.80272500	0.84729500	1.32106400
C	-0.72160900	-0.28881200	0.34709800
H	-0.66706300	-1.20653600	0.93591000
C	-1.85856200	0.01215800	-0.28069600
H	-1.95388600	0.91389900	-0.88202600

H	-2.73555300	-0.62565200	-0.21721800

4a

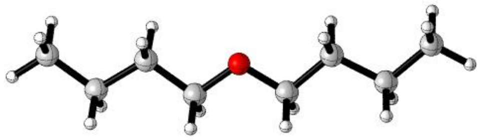

0 1

C	4.92265400	0.55536500	0.00000000
H	5.86986100	0.00615500	-0.00000100
H	4.91127900	1.20338300	0.88379000
H	4.91127200	1.20338100	-0.88379000
C	3.72144600	-0.39524400	0.00000500
H	3.77460300	-1.05368500	0.87791600
H	3.77461100	-1.05370300	-0.87789300
C	2.38035400	0.34663900	-0.00000500
H	2.31018600	0.99924500	-0.87935100
H	2.31017900	0.99925600	0.87933200
C	1.18368400	-0.59448500	-0.00000100
H	1.21464100	-1.25326300	0.88691900
H	1.21463700	-1.25326600	-0.88691900
O	0.00000000	0.18221200	0.00000000
C	-1.18368400	-0.59448500	-0.00000200
H	-1.21463800	-1.25326700	0.88691600
H	-1.21463900	-1.25326200	-0.88692200
C	-2.38035400	0.34663900	0.00000100
H	-2.31018200	0.99925300	-0.87933900
H	-2.31018200	0.99924800	0.87934400
C	-3.72144600	-0.39524400	-0.00000100

H	-3.77460700	-1.05369700	0.87790200
H	-3.77460700	-1.05369200	-0.87790700
C	-4.92265400	0.55536500	0.00000200
H	-4.91127600	1.20338500	-0.88378600
H	-5.86986100	0.00615500	0.00000100
H	-4.91127500	1.20337900	0.88379400

Br^-

-1 1

Br	0.00000000	0.00000000	0.00000000

H_2O

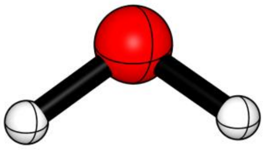

0 1

O	0.00000000	0.00000000	0.11884100
H	0.00000000	0.76012300	-0.47536500
H	0.00000000	-0.76012300	-0.47536500

H_3O^+

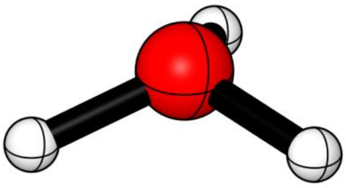

1 1

O	0.00000000	0.00000000	0.07010100
H	0.00000000	0.94749600	-0.18693600

H	-0.82055600	-0.47374800	-0.18693600
H	0.82055600	-0.47374800	-0.18693600

HBr

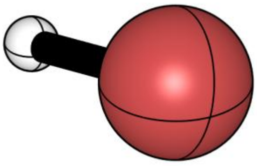

0 1

Br	0.00000000	0.00000000	0.03953700
H	0.00000000	0.00000000	-1.38378300

int1

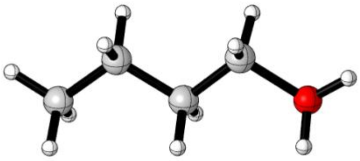

1 1

C	-2.57660900	-0.33610300	-0.01207600
H	-3.47145400	0.29017800	0.01881000
H	-2.61342200	-1.01657900	0.84455000
H	-2.61701100	-0.93520600	-0.92710200
C	-1.31756500	0.53706600	0.02480600
H	-1.31321800	1.14787400	0.93562200
H	-1.31852400	1.22941500	-0.82543100
C	-0.03412700	-0.32764500	-0.02376400
H	-0.02059900	-0.92249100	-0.94309900
H	-0.03215600	-1.01395600	0.83362900
C	1.17399100	0.56588800	0.02352900

H	1.29600200	1.12247200	0.95458900
H	1.29410000	1.20619800	-0.85038700
O	2.44870100	-0.34679900	-0.08120900
H	3.30039900	0.12735200	-0.01228600
H	2.43213600	-1.09609700	0.54580300

int2

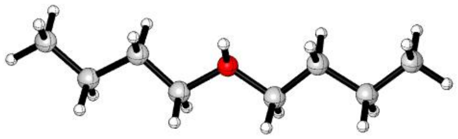

1 1

C	0.56509200	-0.00508900	-4.98605300
H	0.01540100	-0.03592800	-5.93004400
H	1.23795400	-0.86848600	-4.96192000
H	1.17688300	0.90270900	-4.98359900
C	-0.40461900	-0.02341400	-3.80007500
H	-1.02568100	-0.92682600	-3.84037700
H	-1.08691300	0.83305300	-3.86371600
C	0.34442900	0.02491700	-2.45201100
H	0.95069600	0.93543400	-2.39247000
H	1.02313200	-0.83713000	-2.38657000
C	-0.63518100	-0.00450500	-1.30129400
H	-1.22439400	-0.92413100	-1.24687500
H	-1.28669500	0.86944200	-1.26620000
O	0.13901500	0.09153200	0.00000000
C	-0.63518100	-0.00450500	1.30129400
H	-1.22439400	-0.92413100	1.24687500
H	-1.28669500	0.86944200	1.26620000
C	0.34442900	0.02491700	2.45201100

H	0.95069600	0.93543400	2.39247000
H	1.02313200	-0.83713000	2.38657000
C	-0.40461900	-0.02341400	3.80007500
H	-1.02568100	-0.92682600	3.84037700
H	-1.08691300	0.83305300	3.86371600
C	0.56509200	-0.00508900	4.98605300
H	1.17688300	0.90270900	4.98359900
H	0.01540100	-0.03592800	5.93004400
H	1.23795400	-0.86848600	4.96192000
H	0.89046300	-0.53143700	0.00000000

TS1

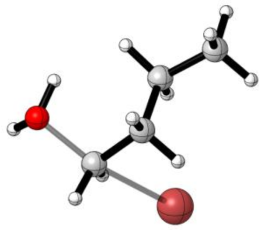

0 1

C	2.13625700	-2.25038200	-0.08781000
H	2.30594200	-2.83603600	-0.99576300
H	1.38577300	-2.77389300	0.51276200
H	3.07305700	-2.22553400	0.48080900
C	1.63886400	-0.84318000	-0.42438500
H	0.67708400	-0.89455700	-0.94824800
H	2.37667600	-0.37086600	-1.10392500
C	1.42854300	0.01161000	0.83575700
H	2.35205100	0.14159200	1.41370700
H	0.69591900	-0.50076300	1.47269000
O	1.85399400	2.17078200	-0.43953800
H	1.32476200	2.77269500	-0.99090400

H	2.21895900	1.48683500	-1.03492000
C	0.75865500	1.32126800	0.56037400
H	0.70167400	2.02232900	1.39132500
H	-0.16538500	1.22648300	-0.04006400
Br	-1.93006800	-0.13858400	-0.05556500

TS2

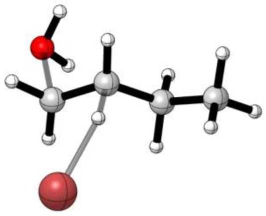

0 1

C	-1.30103000	2.69156100	0.05212300
H	-1.70366800	3.41972800	-0.65810400
H	-0.22137600	2.85043900	0.13187600
H	-1.74663900	2.89627800	1.03221700
C	-1.59321100	1.25854300	-0.40059700
H	-1.13106500	1.07874100	-1.37898200
H	-2.67622600	1.11373000	-0.52588900
C	-1.05283100	0.22444500	0.58186400
H	-1.35926700	0.34896000	1.62351400
H	0.15533300	0.34766700	0.57955400
O	-2.99643100	-1.99910700	-0.13134300
H	-3.33100100	-1.59722300	-0.94476700
H	-3.50066000	-1.57646200	0.57723000
C	-0.85908900	-1.10806000	0.15772600
H	-0.64315900	-1.90295800	0.86104500
H	-0.68290400	-1.33795400	-0.88554000
Br	1.98997300	-0.22991500	-0.04880300

TS3

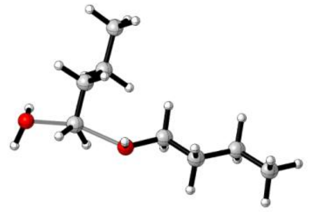

1 1

C	-2.39351300	2.69366500	0.19071100
H	-2.43463900	3.46624900	-0.58083600
H	-1.57948800	2.94885700	0.87753400
H	-3.33135400	2.73118400	0.75450400
C	-2.17314500	1.31220600	-0.43060900
H	-1.24131600	1.30271600	-1.00879600
H	-2.97820200	1.10034500	-1.14837000
C	-2.11589300	0.20537500	0.63181600
H	-3.03667200	0.16784700	1.22717900
H	-1.32076800	0.41767300	1.35627300
O	0.30914000	-1.29626700	0.43021700
C	1.17333500	-0.23883600	-0.06429700
H	0.89101100	0.71960200	0.39394200
H	0.96171800	-0.17857300	-1.13610000
C	2.64141900	-0.55305800	0.18645200
H	2.87661900	-1.52556400	-0.26128700
H	2.81086800	-0.64853100	1.26901700
C	3.56941900	0.53292800	-0.37958000
H	3.30884500	1.50300100	0.06380100
H	3.39603000	0.62854800	-1.45936800
C	5.04753900	0.22649900	-0.11940900
H	5.34059800	-0.72360900	-0.57838700
H	5.68817800	1.00978500	-0.53362200
H	5.25420800	0.15805700	0.95400500

O	-3.71383400	-1.58262200	-0.44310100
H	-3.79916900	-2.42707400	-0.91427800
H	-4.10100800	-0.90241900	-1.01670200
C	-1.85645900	-1.16277200	0.09421000
H	-1.87341100	-2.02511900	0.74711700
H	-1.49411900	-1.31641900	-0.91285500
H	0.54340000	-1.47148500	1.35453800

TS4

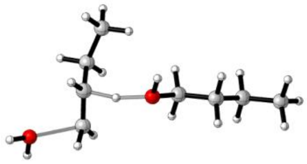

1 1

C	-1.54335700	2.55445200	-0.12972700
H	-1.54671400	3.29308900	-0.93445500
H	-0.50251500	2.39205000	0.16956900
H	-2.07369900	2.98993400	0.72337700
C	-2.20563000	1.25096400	-0.58759300
H	-1.69371900	0.84144500	-1.46690200
H	-3.24006400	1.44427200	-0.89626500
C	-2.25985700	0.19696500	0.50716000
H	-2.63054500	0.53510600	1.47839000
H	-1.02002400	-0.10449600	0.78538200
O	0.37852500	-0.09342200	1.09998900
C	1.31152100	-0.06385400	-0.02925000
H	1.17972600	0.87423900	-0.58146200
H	0.99461700	-0.89220900	-0.66863100
C	2.74528300	-0.24557000	0.44113100

H	2.81460800	-1.17301000	1.02119500
H	3.01119300	0.57879900	1.11816900
C	3.73412400	-0.27790200	-0.73530700
H	3.63891900	0.64771100	-1.31777100
H	3.46433700	-1.09983800	-1.41110400
C	5.18293600	-0.44784300	-0.26785900
H	5.31040100	-1.38017700	0.29232500
H	5.86792900	-0.47273400	-1.11961900
H	5.48792900	0.37875400	0.38267200
O	-4.49160400	-1.53694300	-0.51501400
H	-5.17661100	-1.60709200	0.16398400
H	-4.67153100	-2.25605000	-1.13656000
C	-2.36128900	-1.15245100	0.24333500
H	-2.50947400	-1.87353200	1.04080000
H	-2.05315200	-1.54634300	-0.72094600
H	0.63063300	0.58444000	1.74670600

2. The potential energy profiles of the competitive reaction pathways (at 373 K)

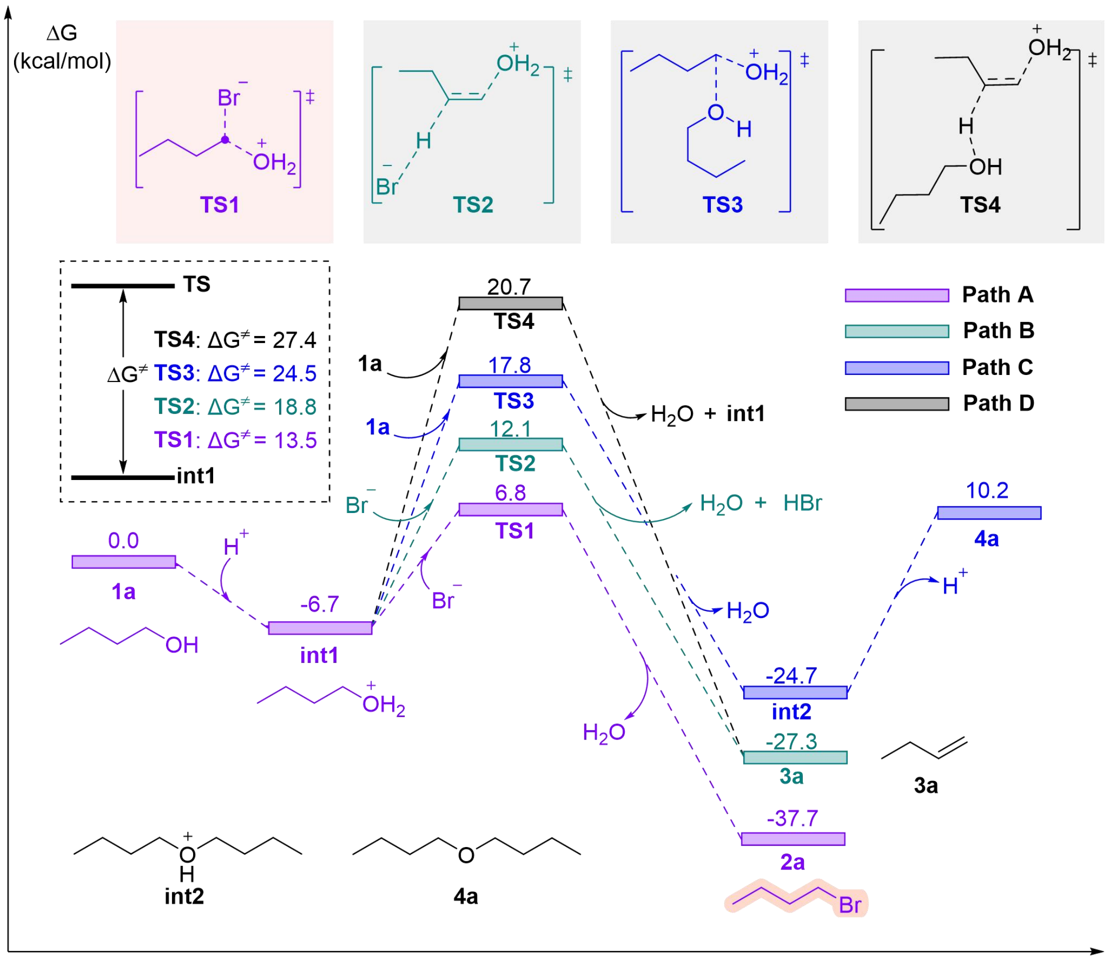

Figure S1. The potential energy profiles of the competitive reaction pathways (at 373 K).

3. A step-by-step hands-on tutorial for the S_N2 pathway (Path A)

Example S_N2 Pathway of n-Bromobutane Synthesis as follow:

3.1 Level of Theory

(1) Functional: B3LYP with Grimme's D3 dispersion correction. (2) Basis set for optimization: 6-31G(d,p). (3) Basis set for single-point energy: 6-311++G(d,p). (4) Solvation model: SMD with n-butanol as solvent.

Note to students: B3LYP is a hybrid density functional that provides good accuracy for organic molecules. The 6-31G(d,p) basis set adds polarization functions (d on heavy atoms, p on H), improving the description of bonding. The 6-311++G(d,p) basis set is larger and more accurate (triple-zeta with additional diffuse functions), used for final energy refinement because it better describes electron density far from

nuclei. SMD is an implicit solvent model that simulates the n-butanol environment without explicitly adding solvent molecules. Grimme's D3 dispersion correction accounts for long-range van der Waals interactions, which are important in transition state structures with partially formed bonds.

3.2 Input File Preparation

Before attempting any transition state (TS) search, we must first obtain the optimized structures of all minima (reactants, intermediates and products) on the potential energy surface.

Step 1: Draw the molecule in GaussView:

(1) Open GaussView. Go to File→New→Create MolGroup. (2) Use the Builder panel to draw the molecular structure. You can draw n-butanol by selecting carbon, oxygen and hydrogen atoms from the Element palette, or import coordinates from an existing .xyz file. (3) After drawing, go to Calculate→Gaussian. Use Clean to roughly adjust bond lengths and angles. (4) Set the route section:

Job Type: Optimization (choose "Optimization")

Method: DF: B3LYP

Basis set: 6-31G(d,p)

Solvation: Select SMD and set n-butanol as the solvent

Additional Keywords: *EmpiricalDispersion=GD3*

(5) Save the file as n-butanol_opt.com.

If you already have the coordinates (e.g., from a .xyz file), you can also create the input file directly using a text editor.

Step 2: Transition state guess structure

To locate TS1 (S_N2 transition state), use the drawing tools to place a Br^- at an appropriate position (approximately 2.0–2.5 Å from the α-carbon) for backside attack on the protonated hydroxyl group. Alternatively, generate a linear interpolation between reactant and product geometries using QST2 or a stepwise scan.

3.3 Geometry Optimization

Once GaussView is set up, let’s run a geometry optimization. #p opt freq b3lyp/6-31g(d,p) empiricaldispersion=gd3 scf=xqc 5d 7f. 6-311++G(d,p) for single-point energy calculations), solvation model (SMD with n- butanol as the solvent). #p b3lyp/6-311++g(d,p) 5d 7f empiricaldispersion=gd3 scrf=(smd,solvent=1-butanol) ExternalIteration,dovacuum.

Input Breakdown:

#p – Print additional information in output file.

opt – Perform geometry optimization to a stationary point.

freq – Calculate vibrational frequencies for the optimized structure. Useful (i) to confirm the stationary point is a minimum (all positive frequencies) and (ii) to obtain thermochemical corrections such as zero-point energy, enthalpy and Gibbs free energy.

b3lyp/6-31g(d,p) – Level of theory for DFT calculation.

scrf=(smd,solvent=1-butanol) – Implicit solvation model with n-butanol as solvent. Note that n-butanol is often specified as 1-butanol in the input file syntax.

EmpiricalDispersion=GD3 – Activate Grimme's D3 dispersion correction.

Explanation for students: A geometry optimization finds the arrangement of atoms where the molecular energy is at a minimum. The opt keyword causes Gaussian to move atoms iteratively along the direction of steepest descent on the potential energy surface. The freq keyword then calculates the second derivatives of energy, yielding the vibrational frequencies. Positive frequencies confirm that the optimized structure is a true local minimum (no uphill curvature in any direction).

3.4 Running Frequencies for Minima

Follow these steps to verify your optimized structure: (1) Save the calculation output file. (2) Once the calculation is complete, open the .log file in GaussView. (3) Go to Results →Vibrations. (4) Examine the frequencies listed. All frequencies should be positive. If negative (“imaginary”) frequencies appear, the optimization has not reached a true minimum. Negative frequencies indicate that the geometry was optimized to a saddle point (transition state) or that the structure is vibrationally

unstable. (5) Under Results→Summary, you can also view the calculated thermochemical parameters: zero-point energy (ZPE), enthalpy (H) and Gibbs free energy (G).

3.5 Transition State Search (S_N2 Pathway)

Step A: Prepare TS guess structure: (1) Open the optimized neutral n-butanol structure in GaussView. (2) Modify the molecule: Change the OH group to an OH_2^+ group (protonate the oxygen). You can do this by:

Adding an extra hydrogen atom to the oxygen, creating a positively charged OH_2^+ group. This converts the hydroxyl from a poor leaving group (−OH) into an excellent one ($-OH_2^+$).

Placing a bromide ion (Br^-) on the opposite side of the carbon chain relative to the OH_2^+ group (backside position).

Adjusting the Br–C distance to ~2.3 Å as a reasonable starting guess.

(3) Set the appropriate charge and multiplicity: Charge = 0 (The net charge on the system is zero: positively charged OH_2^+ and negatively charged Br^- cancel each other to give a net charge of zero.) Multiplicity = 1 (singlet, all electrons paired).

Step B: Transition State (Opt=TS) Calculation: After preparing a reasonable guess structure for the TS, the next step is to optimize it to a first-order saddle point, which represents the transition state. #p opt=(ts,calcfc,noeigentest) freq b3lyp/6-31g(d,p) EmpiricalDispersion=GD3.

Key Options Explained:

opt=(ts,calcfc,noeigentest) – Optimize to a transition state. Calcfc forces calculation of force constants at the starting point; noeigentest bypasses the internal check for the number of negative eigenvalues (useful if Gaussian incorrectly identifies a large number of negative frequencies during intermediate steps).

freq – Frequency calculation after TS optimization, essential for confirmation of one imaginary frequency.

Explanation for students:

A transition state corresponds to a saddle point on the potential energy surface.

The opt=ts keyword directs Gaussian to locate such a saddle point. The calcfc keyword forces Gaussian to compute force constants at the first optimization step; this helps the algorithm move correctly in the direction of the reaction coordinate. The freq calculation should show exactly one imaginary frequency, whose atomic motion should correspond to the reaction coordinate (Br–C bond forming and C–OH_2^+ bond breaking).

3.6 Checking the Transition State

After the calculation finishes, verify the TS: (1) Open the .log file in GaussView. (2) Go to Results→Vibrations. (3) Look for a negative frequency (listed as a negative number, e.g., -300 cm^{-1}, or represented as "i300"). (4) Animate this negative frequency. The atomic displacements should simultaneously show:

The Br atom moving toward the α-carbon (C–Br bond forming)

The OH_2^+ group moving away from the α-carbon (C–OH_2^+ bond breaking)

(5) Ensure no other negative frequencies exist (only one imaginary frequency means it's a first-order saddle point — the correct TS for a reaction).

If more than one imaginary frequency appears: the TS guess may be far from the true saddle point. Try adjusting the initial geometry, increasing calcall (calculating force constants at every step, more accurate but much more expensive), or optimizing with a lower-level method first (e.g., HF/3-21G) before re-optimizing at B3LYP/6-31G(d,p).

3.7 Intrinsic Reaction Coordinate (IRC) Calculation

An IRC calculation verifies that your TS connects the correct reactants and products along the reaction pathway, rather than some other structure. The IRC traces the minimum energy path downhill from the TS in both directions (toward the reactant and toward the product).

Setup: (1) Open the TS .chk file in GaussView. (2) Go to Calculate→Gaussian. (3) Job Type tab: Select IRC. (4) Set Follow IRC: Both directions (to trace toward both reactants and products). (5) Force constants: Select Calculate once (or Calculate

always if computing resources allow). (6) Number of points (MaxPoints): Increase from default 10 to 30 or 50 to ensure the IRC reaches the minima. (7) Click Submit.

Alternatively, using a text-based input file for IRC: #p irc=(maxpoints=50, calcfc, rcfc) b3lyp/6-31g(d,p) EmpiricalDispersion=GD3.

Note: IRC jobs require initial force constants. The easiest way to provide them is to use the rcfc option, which reads force constants from the checkpoint file generated by the preceding TS frequency calculation.

3.8 Single-Point Refinement

After the IRC confirms that the TS connects the correct minima, we refine the energy using a larger basis set (6-311++G(d,p)).

Why refine with 6-311++G(d,p)?

This triple-zeta basis set includes both polarization and diffuse functions, providing more accurate energetic predictions. The calculation is done as a single-point energy at the B3LYP/DFT level on the previously optimized geometry – no further geometry optimization is performed.

3.9 Energy Extraction and Activation Barrier Calculation

To compare the energy of different stationary points and compute activation energy barriers, we need consistent absolute free energy values from:

Reactant (neutral n-butanol) – after optimization + frequency

Intermediate (protonated intermediate int1) – after optimization + frequency (must be a minimum, all positive frequencies)

Transition state (TS1) – after TS optimization + frequency (must have one imaginary frequency)

Product (n-bromobutane) – after optimization + frequency

Extract Gibbs free energies (ΔG, 298.15 K) from each .log file: (1) Open the .log file in a text editor. (2) Perform a search for "Sum of electronic and thermal Free Energies". (3) Record this value (unit: Hartree). This is the Gibbs free energy at standard temperature (298 K).

Alternatively, you can use the thermochemistry data printed by Gaussian at the end of any frequency calculation job.

Compute activation energy:

ΔG (activation barrier to S_N2) = G(TS1) – G(int1)

where G values are in kcal/mol (conversion: 1 Hartree = 627.51 kcal/mol).

3.10 Visualizing the Reaction Path

After completing the IRC calculation (e.g., 50 points in both directions), you can visualize the entire reaction path: (1) Open the IRC output file (IRC.log) in GaussView. (2) Go to Results→IRC. Use the slider to animate the structural transition from reactants to TS to products. (3) Use Results→Energies to observe the energy change along the reaction coordinate.

3.11 Further Reading and References

Some selected reading and references as follow:

(1) http://bbs.keinsci.com/forum.php

(2) http://sobereva.com/multiwfn/

(3) https://gaussian.com/scrf/?tabid=7

(4) Frisch, M. J. et al. Gaussian 09, Revision E.01. Gaussian, Inc., Wallingford CT, 2013.
